# Integration of Freestanding High‐*k* Oxide Membranes for 2D Ferroelectric Field‐Effect Transistors

**DOI:** 10.1002/advs.202520610

**Published:** 2025-12-14

**Authors:** Zejing Guo, Xuyang Sha, Fang Xu, Guangyi Huang, Jinfeng Zhang, Yang Mou, Guorui Zhao, Jiaqi Liu, Qing Lan, Wenqing Song, Cheng Zhang, Hai Huang, Changlin Zheng, Lingfei Wang, Hangwen Guo, Jian Shen, Wu Shi

**Affiliations:** ^1^ State Key Laboratory of Surface Physics and Institute for Nanoelectronic Devices and Quantum Computing Fudan University Shanghai 200433 China; ^2^ Zhangjiang Fudan International Innovation Center Fudan University Shanghai 201210 China; ^3^ Hefei National Research Center for Physical Sciences at Microscale University of Science and Technology of China Hefei 230026 China; ^4^ State Key Laboratory of Surface Physics and Department of Physics Fudan University Shanghai 200433 China; ^5^ Shanghai Frontiers Science Research Base of Intelligent Optoelectronic and Perception, Institute of Optoelectronic and College of Future Information Fudan University Shanghai 200433 China; ^6^ Hefei National Laboratory Hefei 230088 China

**Keywords:** 2D materials, ferroelectric field‐effect transistors, freestanding oxide membranes, high‐*k* dielectrics, van der Waal integration

## Abstract

Ferroelectric field‐effect transistors (FeFETs) based on oxides with exceptional dielectric and ferroelectric properties offer compelling prospects for energy‐efficient logic, nonvolatile memory, and neuromorphic computing. However, integrating high‐*k* ferroelectrics like BaTiO_3_ (BTO) with semiconductor channels remains challenging due to epitaxial growth constraints and poor complementary metal‐oxide‐semiconductor (CMOS) compatibility. Freestanding ferroelectric membranes offer a promising route for van der Waals (vdW) integration, yet practical implementation is impeded by issues such as mechanical deformation during transfer and defect‐induced leakage. Here, a defect‐tolerant strategy for top‐gate integration of freestanding BTO membranes with MoS_2_ channels is presented. A water‐mediated release combined with polymethyl methacrylate (PMMA)‐assisted transfer process is developed to preserve membrane and interface integrity, while a nanoscale dielectric‐channel junction design effectively suppresses leakage current well below the low‐power limit. The resulting FeFETs exhibit a record‐high memory window of 0.22 V nm^−1^, an ultrahigh dielectric constant of 52, and a near‐ideal subthreshold swing of 60 mV dec^−1^. Furthermore, a fully functional 3 × 4 top‐gate FeFET array enabling in‐memory logic and synaptic functionalities is demonstrated. This work establishes a scalable and transferable platform for incorporating high‑*k* ferroelectric oxides into high‐performance reconfigurable 2D nanoelectronics.

## Graphical abstract

This work introduces a defect‐tolerant integration strategy that enables freestanding BaTiO_3_ membranes to form low‐leakage top‐gate junctions with MoS_2_. The resulting FeFETs exhibit a record 0.22 V nm^−1^ memory window, ultrahigh‐*k* response, and near‐ideal subthreshold swings. A 3 × 4 FeFET array further demonstrates in‐memory logic and synaptic functionality, establishing a scalable platform for incorporating high‐*k* ferroelectric oxides into high‐performance reconfigurable 2D nanoelectronics.

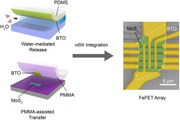

## 1 Introduction

Ferroelectric oxides, characterized by robust spontaneous polarization and reversible electric dipole switching, hold significant potential for next‐generation electronic devices.^[^ ^1^, ^2^, ^3^, ^4^ ^]^ Their exceptional dielectric and ferroelectric properties are particularly advantageous when integrated with 2D layered semiconductors.^[^ ^5^, ^6^, ^7^ ^]^ Such integration could enable compact, multifunctional ferroelectric field‐effect transistors (FeFETs) that are crucial for energy‐efficient logic circuits, non‐volatile memory, and neuromorphic computing systems.^[^ ^8^, ^9^, ^10^, ^11^ ^]^ Perovskite oxides such as barium titanate BaTiO_3_ (BTO) thin films are especially attractive due to their ultrahigh dielectric constants (*ε*_300K_ ≈ 1000) and strong remanent polarization (*P*_r,300K_ = 50 µC cm^−2^),^[^ ^2^, ^12^ ^]^ which are critical for high‐performance FeFET operations. However, direct integration of these oxides with semiconductor channel materials via conventional complementary metal‐oxide‐semiconductor (CMOS)‐compatible processes remains difficult, primarily due to substrate‐induced lattice mismatch and thermal incompatibility.^[^ ^13^, ^14^ ^]^

Recent advances in synthesizing freestanding oxide membranes offer a promising route for van der Waals (vdW) integration with 2D semiconductors, circumventing these substrate‐related constraints.^[^ ^15^, ^16^, ^17^, ^18^, ^19^, ^20^ ^]^ Notably, freestanding ferroelectric membranes not only retain their ferroelectric properties but also exhibit superelasticity, making them highly desirable for flexible electronics.^[^ ^21^, ^22^, ^23^ ^]^ Despite these advantages, several critical challenges remain to hinder their practical implementation in FeFETs. For example, the inherent elasticity of freestanding membranes often leads to curling during transfer and integration, compromising interfacial integrity.^[^ ^24^ ^]^ More importantly, intrinsic defects, particularly oxygen vacancies, can introduce significant leakage currents, which mask or disrupt genuine ferroelectric switching.^[^ ^18^, ^25^, ^26^, ^27^ ^]^ Conventional attempts to mitigate leakage by incorporating dielectric buffer layers frequently weaken polarization coupling and introduce charge‐trapping hysteresis rather than enabling true ferroelectric behavior. Therefore, achieving reliable, low‐leakage and CMOS‐compatible integration of freestanding ferroelectric oxide membranes with 2D materials remains a major challenge.

Here, we achieve high‐performance FeFETs by introducing a defect‐tolerant strategy to integrate high‐*k* freestanding BTO membranes as top‐gate dielectrics with MoS_2_ channels. By combining an anti‐curling fabrication technique based on water‐mediated release and polymethyl methacrylate (PMMA)‐assisted transfer with a miniaturized dielectric‐channel junction design, we establish high‐quality vdW interfaces and greatly suppress leakage currents in BTO/MoS_2_ heterostructures. Consequently, these devices achieve robust ferroelectric switching with a record‐high memory window (MW) of 0.22 V nm^−1^. They also exhibit an ultrahigh dielectric constant of 52, an ideal subthreshold swing (SS) of 60 mV dec^−1^, demonstrating mechanical flexibility. Furthermore, our approach is scalable as demonstrated by a fully functional 3 × 4 FeFET array with compact geometries, supporting both in‐memory logic and programmable synaptic operations. Our integration approach establishes a versatile platform for implementing ferroelectric oxide membranes in high‐performance 2D nanoelectronics.

## 2 Van der Waals (vdW) Integration of Ferroelectric BTO Membranes with 2D Materials

**Figure 1:**
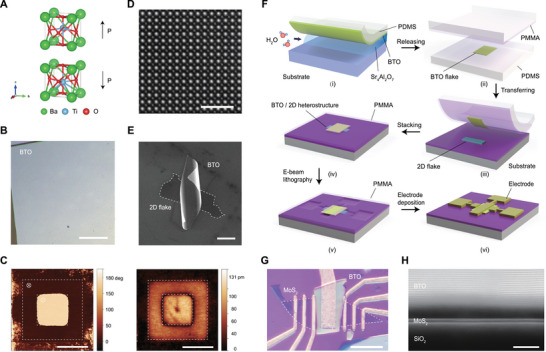
vdW integration of ferroelectric BTO membranes with 2D materials. A) Crystal structure of perovskite ferroelectric BTO, illustrating up (top) and down (bottom) polarization states. B) Optical image of a large‐scale PDMS‐supported freestanding BTO membrane released from the water‐soluble SAO_T_ sacrificial layer. Scale bar, 200 µm. C) Domain reversal in a BTO membrane with a tip bias of ±9 V, shown in phase (left) and amplitude (right) images. Scale bar, 1 µm. D) Plane‐view HADDF‐STEM image of a transferred freestanding BTO membrane, revealing well‐ordered atomic arrangements. Scale bar, 2 nm. E) SEM image of a curled BTO membrane/graphene heterostructure fabricated via standard PC‐assisted dry‐transfer, illustrating the membrane's elasticity. Scale bar, 10 µm. F) Schematic of the anti‐curling fabrication technique. Dissolving the SAO_T_ layer in deionized water releases the large‐scale BTO membrane, which adheres to PDMS. By repetitive laminating and peeling‐off by another PDMS, micrometer‐sized BTO flake is obtained and then transferred by a PDMS/PMMA stamp. The PMMA layer also serves as an electron‐beam resist for electrode patterning. Finally, metal electrodes confine and flatten the membrane. G) Optical image of a BTO/MoS_2_ heterostructure using BTO membrane as the top‐gate dielectric. The dashed line outlines the MoS_2_ flake. Scale bar, 10 µm. H) Cross‐sectional HAADF‐STEM image of the BTO‐bilayer MoS_2_ heterointerface in the transistor channel region, showing a uniform vdW gap. Scale bar, 5 nm.

BTO thin films exhibit a tetragonal phase with large room‐temperature ferroelectricity (**Figure** **1**A), as evidenced by characteristic polarization‐electric field hysteresis loops (Figure S1, Supporting Information). They also possess high dielectric constant, as confirmed by our thickness‐dependent capacitance measurements (Figure S2, Supporting Information). By using a newly developed water‐soluble Sr_4_Al_2_O_7_ (SAO_T_) sacrificial layer, we can fabricate large‐scale, high‐quality freestanding BTO membranes.^[^ ^22^ ^]^ The low‐symmetry crystal structure of SAO_T_ supports coherent epitaxial growth of BTO heterostructures (Figure S3, Supporting Information), enabling damage‐free membrane release while preserving excellent crystallinity. As shown in Figure 1B, this process produces crack‐free BTO membranes spanning hundreds of micrometers.

We confirm the ferroelectric properties of the released BTO membranes through piezoresponse force microscopy (PFM), which demonstrates stable domain switching under ±9 V bias (Figure 1C). Bright and dark regions in the phase image correspond to out‐of‐plane ferroelectric domains with upward and downward polarization, respectively, while the dark square in the amplitude image indicates domain wall. Phase hysteresis loops and butterfly‐shaped amplitude responses (Figure S4, Supporting Information) further validate robust out‐of‐plane polarization. Atomic‐resolution high‐angle annular dark‐field scanning transmission electron microscopy (HAADF‐STEM) imaging (Figure 1D) reveals well‐ordered atomic arrangements, confirming the structural integrity of the transferred membranes.

Although the superelastic nature of these membranes benefits flexible electronics, it often causes severe curling due to stress release during membrane separation and conventional polycarbonate (PC)‐assisted transfer (Figure S5, Supporting Information), creating significant integration challenges. As shown by the scanning electron microscopy (SEM) image of a curled BTO/graphene heterostructure (Figure 1E) fabricated with conventional PC‐assisted transfer, such curling leads to non‐uniform vdW interfaces that impede reliable device fabrication. To address this, we develop an anti‐curling fabrication approach that combines three key steps: water‐mediated release, PMMA‐assisted transfer, and metal electrode confinement. During release and transfer, a polydimethylsiloxane (PDMS) support layer maintains membrane flatness while the SAO_T_ layer dissolves, and the viscoelastic PMMA stamp counteracts bending stress during vdW assembly with 2D materials. PMMA also functions as an electron‐beam resist for electrode patterning. Finally, metal electrodes are designed to confine the membrane and apply tensile stress to flatten it, yielding wrinkle‐free vdW interfaces. This process is illustrated in Figure 1F and detailed in the Experimental Section. Optical microscopy of the resulting BTO/MoS_2_ heterostructure (Figure 1G) confirms successful edge clamping, while cross‐sectional STEM image shows a uniform vdW gap between the BTO and MoS_2_ channel (Figure 1H; Figure S6, Supporting Information). Overall, this integrated approach enables both large‐area processing and high‐quality interfaces in a scalable manner.

## 3 Nanoscale Dielectric‐Channel Junctions for Leakage Mitigation

**Figure 2:**
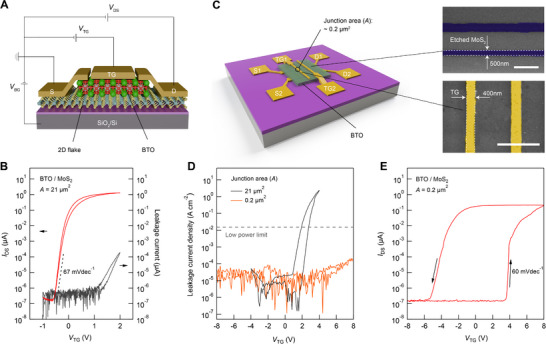
Leakage current mitigation in top‐gate BTO/MoS_2_ transistors. A) Schematic of the dual‐gate 2D transistor with freestanding BTO membrane as the top‐gate dielectric, illustrating the electrical measurement configuration. S, source; D, drain; TG, top gate. B) Source‐drain current *I*_DS_ and top‐gate leakage current versus top‐gate voltage *V*_TG_ for a BTO/MoS_2_ transistor with a dielectric‐channel junction area (*A*) of 21 µm^2^. C) Schematic of the defect‐tolerant design, featuring a significantly miniaturized junction area (0.2 µm^2^). The MoS_2_ flakes are nanoscale‐patterned into ≈500nm‐wide ribbons to form the channel (top right), and the top‐gate electrodes are ≈400 nm wide (bottom right). Scale bar, 2 µm. D) Leakage current density versus *V*_TG_ of two BTO/MoS_2_ devices with junction area of ≈21 µm^2^ (black) and ≈0.2 µm^2^ (orange). The miniaturized junction shows a substantially lower leakage current density well below the low power limit. E) Transfer curve *I*_DS_–*V*_TG_ for a BTO/MoS_2_ transistor with a 0.2 µm^2^ junction area, exhibiting pronounced anticlockwise ferroelectric hysteresis with a memory window of 0.22 V nm^−1^ and a minimal SS of 60 mV dec^−1^.

We first evaluate the performance of BTO/single‐layer MoS_2_ heterostructure as illustrated in **Figure** **2**A, with the freestanding BTO membranes serving as the top‐gate dielectric and a 285 nm SiO_2_ layer functioning as the bottom‐gate dielectric. The MoS_2_ flake is approximately several tens of square micrometers in size. As shown in Figure 2B, when the top‐gate voltage *V*_TG_ is swept from −1 to 2 V, the MoS_2_ channel achieves an on/off ratio exceeding 10^7^ and a near‐ideal SS of 67 mV dec^−1^ (also see Figure S7, Supporting Information). However, such behaviors lack typical ferroelectric‐induced characteristics. The expected ferroelectric hysteresis in the transfer curve is negligible because significant leakage current restricts the operational gate‐voltage range and hinders effective polarization switching (Figure 2B). This leakage likely stems from intrinsic defects such as oxygen vacancies and stress‐induced in‐plane domain walls in freestanding BTO, particularly when the BTO is in direct contact with metal electrodes and the channel material over large areas (Figure ; Figure S8, Supporting Information). These defects have commonly prevented 2D material channels from exhibiting pronounced anticlockwise ferroelectric hysteresis.^[^ ^18^, ^26^, ^27^ ^]^

To overcome these limitations, we adapted a miniaturized dielectric‐channel junction design to mitigate the leakage current. We used the same freestanding BTO membranes without additional growth optimization to address their intrinsic defect densities. As depicted in Figure 2C, the BTO membrane was transferred via the anti‐curling approach onto single‐ or few‐layer MoS_2_ strips (≈500 nm‐wide) that were nanoscale‐patterned via reactive ion etching (RIE). The top‐gate electrodes, with ≈400 nm in width, were oriented perpendicular to the MoS_2_ channels to form an array. In this configuration, each junction area between the BTO dielectric and MoS_2_ channel (≈0.2 µm^2^) is reduced by two orders of magnitude compared to the design in Figures 1G and 2A,B. This defect‐tolerant miniaturized design significantly diminishes leakage current by minimizing interfacial defect pathways and helps suppressing depolarization effects. As shown in Figure 2D, the device can operate over an extended gate‐voltage range from −8 to +8 V while keeping the leakage current density well below the low power limit.

With this optimized architecture, we observe clear ferroelectric switching characteristics in a 40 nm‐BTO/MoS_2_ transistor, evidenced by wide and sharp anticlockwise hysteresis in bidirectional transfer curves (Figure 2E). This intrinsic polarization switching produces a substantial memory window of 0.22 V nm^−1^ and a minimal SS of 60 mV dec^−1^. These results confirm that our strategy effectively mitigates leakage current, thereby enabling robust ferroelectric gating. Consequently, this integration approach provides a promising platform for realizing high‐performance 2D FeFETs with freestanding ferroelectric BTO membranes.

## 4 High‐Performance Device Characteristics of Top‐Gate BTO/MoS_2_ Transistors

**Figure 3:**
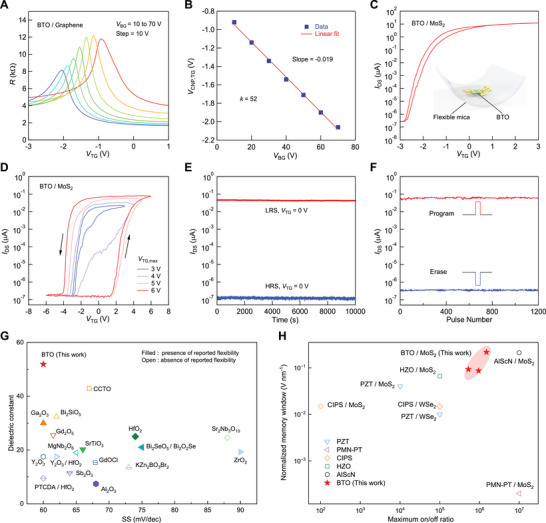
Device characteristics of top‐gate BTO/MoS_2_ transistors. A) Sheet resistance *R* of a BTO/graphene transistor as a function of *V*_TG_ at different *V*_BG_ from 10 to 70 V (right to left). B) Top‐gate charge neutrality point (CNP) of graphene *V*_CNP, TG_ as a function of *V*_BG_, extracted from A. The red solid line is a linear fit with a slope of −0.019, yielding a BTO dielectric constant of ≈52. C) *I*_DS_–*V*_TG_ characteristics of a BTO/MoS_2_ transistor on a transparent, flexible mica substrate. Inset is illustration of a flexible BTO/MoS_2_ transistor on mica. D) Transfer curves of a BTO/MoS_2_ FeFET at *V*_DS_ = 0.5 V under various top‐gate sweep ranges, showing a maximum memory window ≈56% at ±6 V. E) Retention characteristic of the device in D, illustrating stable high (blue curve) and low (red curve) resistance states at *V*_TG_ = 0 V for over 10^4^ s with an on/off ratio exceeding 10^5^. F) Endurance performance evaluated over 1200 cycles using +6 V (1s) program and −6 V (1s) erase pulses. G) Comparison of dielectric constant and SS for our BTO/MoS_2_ transistors with previously reported top‐gated MoS_2_ FETs (cyan solid dot for Bi_2_SeO_5_/Bi_2_O_2_Se) using various high‐*k* dielectrics.^[^ ^15^, ^20^, ^28^, ^29^, ^30^, ^31^, ^34^, ^35^, ^36^, ^37^, ^38^, ^39^, ^40^, ^41^, ^42^, ^43^, ^44^, ^45^, ^46^, ^47^ ^]^ Filled symbols indicate reported mechanical flexibility, while open symbols indicate the absence of reported mechanical flexibility. H) Comparison of normalized memory window and maximum on/off ratios for our BTO/MoS_2_ FeFETs with previously reported 2D FeFETs with the same metal‐ferroelectric‐semiconductor architectures.^[^ ^48^, ^49^, ^50^, ^51^, ^52^, ^53^, ^54^ ^]^ Red stars represent our MoS_2_ FeFETs with different BTO thickness of 40, 55, and 72 nm.

Having addressed the leakage current issue through the defect‐tolerant strategy, we next systematically evaluate the device performance of these compact top‐gate BTO/MoS_2_ transistors. First, we determine the effective dielectric constant of the freestanding BTO membrane using dual‐gate measurements on a graphene channel (**Figure** **3**A).^[^ ^20^ ^]^ In these experiments, *V*_TG_ is swept within a low‐voltage range that does not induce polarization switching in the BTO dielectric. We then analyze the shifts in the top‐gate charge neutrality point (CNP) as a function of the back‐gate voltage *V*_BG_ (Figure 3B). According to a parallel‐plate capacitor model, the slope of the CNP shift with respect to *V*_BG_ is correlated to the capacitance ratio of the bottom and top‐gate dielectrics, given by:

(1) $$\frac{C_{{\mathrm{SiO}}_{2}}}{C_{\mathrm{BTO}}}=\frac{\varepsilon_{{\mathrm{SiO}}_{2}}t_{\mathrm{BTO}}}{\varepsilon_{\mathrm{BTO}}t_{{\mathrm{SiO}}_{2}}}\;$$

where $\varepsilon_{{\mathrm{SiO}}_{2}}$ and $\varepsilon_{\mathrm{BTO}}$ represent the effective dielectric constants of SiO_2_ and BTO, respectively, and $t_{{\mathrm{SiO}}_{2}}$ and $t_{\mathrm{BTO}}$ are their respective thicknesses (285 nm for SiO_2_ and 72 nm for BTO). Substituting the measured slope into this formula yields an effective dielectric constant of 52 for BTO, surpassing those of well‐known high‐*k* dielectrics such as HfO_2_, Bi_2_SeO_5_, and freestanding SrTiO_3_.^[^ ^15^, ^20^, ^28^, ^29^, ^30^, ^31^ ^]^ Notably, the effective dielectric constant of the transferred membrane is lower than that of the as‐grown epitaxial film (Figure S2, Supporting Information). This reduction is attributed to strain relaxation and parasitic capacitance arising from non‐ideal van der Waals interfaces.^[^ ^20^, ^32^ ^]^ This suggests that the high performance achieved here does not yet represent BTO's intrinsic limit, leaving room for further enhancement. Future improvements in transfer and interface engineering are expected to mitigate these extrinsic effects and fully realize high permittivity.

Beyond ultrahigh dielectric constant, the BTO/MoS_2_ devices also exhibit mechanical flexibility, enabling high device performance on both rigid and flexible substrates. To demonstrate this capability, we fabricated MoS_2_ transistors on flexible mica substrates using BTO membrane as the top‐gate dielectric. These devices are highly transparent and bendable, as illustrated in the inset of Figure 3C and Figure S9 (Supporting Information). Operating at a low gate‐voltage range, this flexible BTO/MoS_2_ transistor exhibits an on‐off ratio approaching 10^8^ at *V*_DS_ = 0.5 V in the flat state (Figure 3C). Notably, the device maintains consistent performance under both tensile and compressive bending (Figure S9, Supporting Information).

We further investigate the performance of BTO/MoS_2_ transistors by expanding the gate‐voltage sweep range to induce ferroelectric switching. As shown in Figure 3D, a 72 nm BTO‐based MoS_2_ FeFET yields a memory window exceeding 55.7% as the total sweep range increases to ±6 V. Moreover, in this dual‐gate FeFET configuration, the memory window can be effectively shifted by applying back‐gate voltages (Figure S10, Supporting Information). The thermal stability of the ferroelectric switching behavior was also investigated by measuring the temperature‐dependent transfer characteristics (Figure S11, Supporting Information). The hysteresis gradually narrows with increasing temperature and recovers upon cooling to room temperature. This near‐complete restoration of ferroelectric hysteresis loop provides strong evidence that the observed MW reduction is primarily driven by the intrinsic ferroelectric‐paraelectric phase transition.^[^ ^18^, ^33^ ^]^ While we do observe minor indications of temperature‐activated defects evidenced by a slight increase in the off‐state current after thermal cycling, these effects are clearly secondary. The essential ferroelectric switching behavior is well‐preserved at temperatures exceeding 100 °C, supporting its potential for practical applications.

To assess the reliability of the BTO‐based MoS_2_ FeFETs for memory applications, we examined their stability and retention characteristics. As shown in Figure 3E, two distinct high‐ and low‐resistance states are maintained when the gate voltage returns to 0 V, with binary retention tests demonstrating stability for over 10^4^ s and an on/off ratio exceeding 10^5^. Furthermore, applying periodic ±6 V pulse voltages (1 s duration, separated by 10 s intervals) reliably toggles the channel current between the two states. Over 1200 cycles, both resistance states showed minimal degradation, consistently maintaining an on/off ratio close to 10^5^ (Figure 3F). This operational endurance is complemented by the consistent transfer curves over multiple operation cycles (Figure S12, Supporting Information), which demonstrates cycle‐to‐cycle repeatability of the switching characteristic. We have evaluated the FeFETs with different BTO thicknesses (40 nm in Figure 2E and 72 nm in Figure 3D; 55 nm in Figure S13, Supporting Information), and all devices exhibited pronounced anticlockwise hysteresis compatible with inherent ferroelectric switching behavior (Figure S14, Supporting Information), underscoring the robustness of our integration strategy.

We compare our transistors with state‐of‐the‐art MoS_2_ transistors utilizing other high‐*k* oxide dielectrics, in terms of dielectric constant, SS, and mechanical flexibility.^[^ ^15^, ^20^, ^28^, ^29^, ^30^, ^31^, ^34^, ^35^, ^36^, ^37^, ^38^, ^39^, ^40^, ^41^, ^42^, ^43^, ^44^, ^45^, ^46^, ^47^ ^]^ As shown in Figure 3G, our BTO‐integrated transistors demonstrate an ultrahigh dielectric constant and superior overall performance. Furthermore, we compare the normalized memory window and maximum on/off ratio of our BTO‐based 2D FeFETs with previous reports on 2D FeFETs for memory applications (Figure 3H; Table S1, Supporting Information).^[^ ^48^, ^49^, ^50^, ^51^, ^52^, ^53^, ^54^ ^]^ Normalizing the memory window by the ferroelectric thickness highlights the robustness of polarization, independent of absolute thickness. This is crucial for device scaling and ensures a fair comparison. While there are variations across devices due to differences in BTO thickness and channel inhomogeneities, our devices consistently rank among the top in both metrics. These results underscore the exceptional potential of freestanding ferroelectric BTO membrane as a flexible high‐*k* ferroelectric for next‐generation 2D logic and memory applications.

## 5 BTO/MoS_2_ FeFET Array with In‐Memory Logic and Synaptic Capabilities

**Figure 4:**
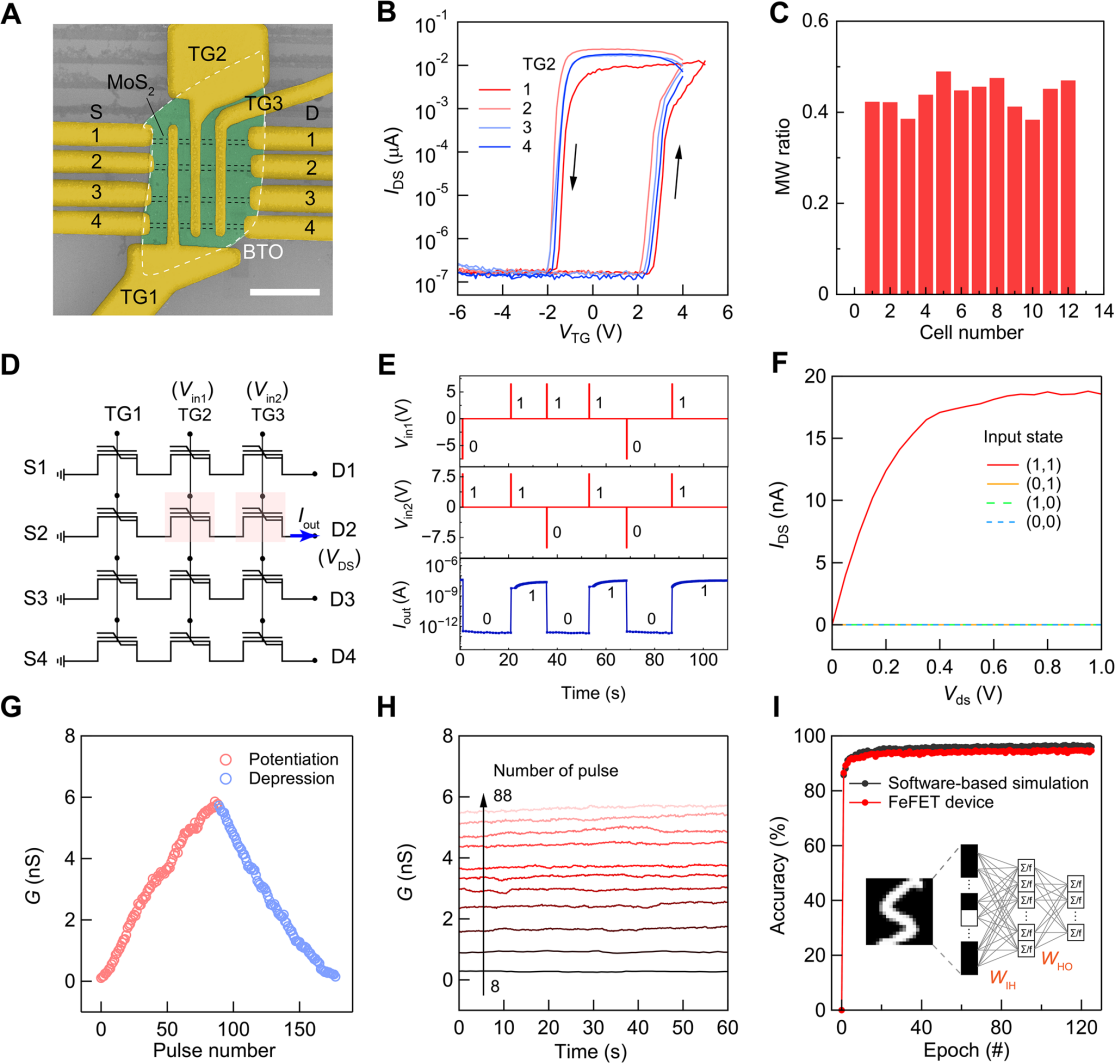
Memory and synaptic operations of top‐gate BTO/MoS_2_ FeFET array. A) False‐color SEM image of a 3 × 4 MoS_2_ FeFET array using freestanding BTO membrane as the top‐gate dielectric. The etched MoS_2_ channels under the BTO membrane are outlined by the black dashed lines. Memory cells in each column share a common top gate, and those in the same row are connected in series. Scale bar, 5 µm. B) Transfer curves of four memory cells that share the second top gate in A. C) Histogram of the memory window (MW) ratios for all 12 memory cells shown in A. D) Schematic illustration of logic AND operations using two memory cells in the same row. *V*_int_, *V*_DS_, and *I*_out_ denote the input voltage, source‐drain voltage, and output current of the AND string, respectively. E) *I*_out_ (lower panel) measured at *V*_DS_ = 1 V after different gate voltage pulse combinations *V*_int1_ and *V*_int2_ (upper panels) are applied successively. F) Output characteristics of the AND strings under four different programming conditions, showing an “on” state only when all memory cells are programmed. G) Synaptic weight update characteristics of a typical FeFET memory cell in the array subjected to a series of 5 ms update pulses, emulating long‐term potentiation (LTP) and long‐term depression (LTD). H) Retention behavior of the excitatory postsynaptic current (EPSC) for input pulse counts ranging from 8 to 88. I) Recognition accuracy as a function of training epochs, based on the LTP/LTD curve in G, reaching a maximum of 94.92% (red curve). Inset is a schematic of a MLP‐based ANN, where *W*_IH_ and *W*_HO_ denote weight of input to hidden layer and weight of hidden layer to output layer, respectively.

Our defect‐tolerant integration strategy also supports the fabrication of functional ferroelectric memory arrays based on BTO/MoS_2_ heterostructures with excellent uniformity. **Figure** **4**A shows a prototype 3 × 4 FeFET array containing 12 independently addressable memory cells, where pre‐patterned MoS_2_ channels (≈500 nm wide) intersect with 400 nm‐wide BTO top gates. This scalable architecture shared source and drain electrodes in each row, while each column has a common gate line. During operation, unaddressed cells maintain “on” state through the intrinsic conduction of MoS_2_, preventing crosstalk during individual cell programming.

We evaluate the array's reliability through transfer characteristics. Figure 4B shows the typical transfer curves of four memory cells sharing the same gate line. All 12 cells display consistent anticlockwise hysteresis loops with a memory window spanning 36–50% of the scan voltage range (Figure 4C; Figure S15, Supporting Information). All memory cells can be reliably programmed or erased using appropriate gate‐voltage pulses and exhibit nonvolatile capabilities. This performance reproducibility, further substantiated by a broader statistical analysis of devices from different arrays (Figure S16, Supporting Information), underscores both defect tolerance and scalability of our approach.

Building on these memory capabilities, we implement reconfigurable logic operations via gate‐level control. The array's architecture inherently supports parallel computing paradigms, and here we demonstrate an AND gate example (Figure 4D–F). Programming adjacent cells in four distinct input pulse configurations produces well‐defined output states corresponding to the standard AND truth table (Figure 4D). Notably, the “0” and “1” outputs exhibit a current ratio exceeding 10^5^ (Figure 4E), which is evident in the *I*_DS_ versus *V*_DS_ curves for different input pulses (Figure 4F). This memory and logic dual functionality highlights the potential for energy‐efficient in‐memory computing.

We further demonstrate the neuromorphic potential of our 2D BTO/MoS_2_ FeFETs by emulating synaptic plasticity, where gate voltage pulses act as presynaptic inputs and the channel conductance serves as the synaptic weight. Figure 4G shows the evolution of the synaptic weight in a typical FeFET memory cell subjected to a series of 5 ms update pulses with gradually increasing amplitudes. The potentiation pulses increase from 5.12 to 6 V in 10 mV steps, while depression pulses decrease from −3.5 to −5.26 V in −20 mV steps. Consecutive positive (or negative) pulses gradually increase (decrease) the synaptic weights with nonlinearity factors of 1.42(1.07), emulating long‐term potentiation (LTP) and long‐term depression (LTD) required for synaptic learning and memory formation. Notably, distinct conductance states can be achieved and retained over the 60 s measurement period (Figure 4H).

Finally, we perform a multilayer perceptron (MLP)‐based ANN simulation using the open‐source code NeuroSimV3.0.^[^ ^55^ ^]^ The network consists of 400 input neurons, 100 hidden neurons, and 10 output neurons, with full connectivity through artificial synapses incorporating the device's nonlinear parameters (see Figure 4I). Training (with 60 000 images) and testing (with 10 000 images) are conducted using the Modified National Institute of Standards and Technology (MNIST) dataset of 20 × 20 pixels handwritten digit images. As shown in Figure 4I, the maximum classification accuracy using our LTP/LTD characteristics reaches 94.92% (vs. 96.65% in software‐based simulations), underscoring the potential of the freestanding BTO‐based FeFET arrays for neuromorphic computing applications.

## 6 Conclusion

In summary, we report a defect‐tolerant vdW assembly of freestanding BTO membranes with MoS_2_, enabling high‐performance FeFETs and functional arrays. By employing an anti‐curling fabrication technique combining water‐mediated release and PMMA‐assisted transfer, along with a miniaturized dielectric‐channel junction design, we realize high‐quality vdW integration and significantly mitigate the leakage currents. The resulting BTO/MoS_2_ transistors demonstrate robust ferroelectric switching with a record‐high memory window of 0.22 V nm^−1^, an ultrahigh‐*k* dielectric constant, and an ideal subthreshold swing. We further demonstrate the scalability of our defect‐tolerant strategy by fabricating a 3 × 4 FeFET array that supports both memory and synaptic operations. Because these high‐quality freestanding oxide membranes can be grown wafer‐scale using the water‐soluble sacrificial layer and integrated in flexible, compact top‐gate geometries, our framework provides a versatile CMOS‐compatible platform for next‐generation large‐scale, energy‐efficient multifunctional logic and memory electronics based on high‐*k* ferroelectric oxides and 2D layered semiconductors. To fully realize this potential in high‐density integration, however, a systematic investigation of short‐channel effects is essential. Future work will therefore focus on analyzing these scaling behaviors to determine the limits of device miniaturization and further optimize integration density.

## 7 Experimental Section

### Growth of Freestanding BTO Membranes and Structure Characterization

BTO and SAO_T_ thin films were grown on (001)‐oriented (LaAlO_3_)_0.3_‐(SrAl_0.5_Ta_0.5_O_3_)_0.7_ [LSAT (001)] single crystalline substrates via pulsed‐laser deposition using a KrF excimer laser (248 nm). During deposition, the substrate temperature was maintained at 700 °C. The SAO_T_ layer was deposited under an oxygen pressure of 0.2 mTorr with a laser fluence of 1.5 J cm^−2^, followed by the BTO layer at 10 mTorr with 1 J cm^−2^. After growth, the heterostructures were prepared for membrane release. A thin sheet of commercial polydimethylsiloxane (PDMS, Gel‐Pak) was used gently brought into conformal contact with the BTO surface, ensuring adhesion via van der Waals forces without applying significant external pressure. The entire PDMS/BTO/SAO_T_/LSAT (001) stack was then immersed in deionized water at room temperature, which selectively etches the sacrificial SAO_T_ layer. Typically, within 30 min, the BTO membrane was fully released from the rigid LSAT substrate, remaining adhered to the PDMS support. This process consistently yields large‐area, crack‐free membranes, which were then carefully retrieved and dried with a gentle stream of nitrogen gas before further transfer. The crystallographic properties of the epitaxial films were characterized using 2*θ*‐*ω* X‐ray diffraction (XRD) scans on a Bruker D8 Discover system (Figure S3, Supporting Information).

### PFM Measurements

PFM measurements were carried out at room temperature using a commercial scanning probe microscope (Dimension Icon, Bruker) equipped with Pt/Ir‐coated atomic force microscopy (AFM) tips (SCM‐PIT‐V2). After dissolving the SAO_T_ layer, the freestanding BTO membrane was transferred onto MoS_2_, where served as the bottom electrode. No top electrode was used during the PFM measurements. PFM hysteresis loops and images were recorded with a 500 mV AC bias applied to the scanning tips, while domain writing was performed by applying ±9 V DC to the tip and scanning the selected regions (Figure 1C; Figure S4, Supporting Information).

### Device Fabrication

For the PMMA‐assisted transfer (Figure 1F), a drop of PMMA (950 A6, Kayaku Advanced Materials, Inc.) was spin‐coated onto the silicon substrate at 1000 rpm for 40 s and baked at 150 °C for 4 min. The resulting PMMA film was picked up using Kapton tape and placed on a PDMS stamp (Sylgard 184, Dow Inc., 10:1 base‐to‐curing agent ratio). When brought into contact with the released BTO membranes on a PDMS support, the viscoelastic PDMS/PMMA stamp picks up the BTO membranes and counteracts their bending stress, allowing them to adhere to its surface without curling. To integrate the BTO with 2D flakes, the chosen flake on SiO_2_/Si was aligned with the BTO membrane on the PMMA stamp using a transfer stage. The substrate was heated to ≈150 °C, releasing the PMMA from the PDMS. Electron beam lithography (EBL) and e‐beam evaporation were then used to pattern the top gate and electrodes, confining the BTO membrane. To fabricate the BTO/MoS_2_ FeFET array, the MoS_2_ flakes were narrowed into ≈400–800 nm ribbons using EBL and RIE. The freestanding BTO membrane was then transferred onto the patterned MoS_2_, and gate electrodes (≈300–500 nm wide) were deposited perpendicular to the underlying MoS_2_ channels. Bi/Au (40/70 nm) was used for all MoS_2_ devices to minimize contact resistance.

### TEM and SEM Characterization

TEM samples were prepared in both plane‐view and cross‐sectional view for structure characterization. The plane‐view samples were prepared by transferring BTO membrane onto a TEM grid with PMMA‐assistance, while the cross‐sectional TEM lamellas were prepared using focused ion beam (Helios G4 CX, Thermo Fisher Scientific) with the standard lift‐out method. The TEM investigations were performed on a field‐emission transmission electron microscope (Themis Z, Thermo Fisher Scientific) equipped with double aberration correctors (SCORR and CETCOR, CEOS GmbH) operating at 300 kV. For HAADF‐STEM imaging, the beam current was set to 40 pA, the semi‐convergence angle was 21.4 mrad, and collection angle was from 63 to 200 mrad. The atomic resolution HAADF‐STEM images were acquired with a fast‐scanning rate (0.25 µs dwell time) to minimize the effects of scan noise and sample drift. 100 frames were integrated using a rigid‐body drift correction algorithm to form the drift‐corrected HAADF‐STEM images. Furthermore, the chemical composition of the interface was qualitatively analyzed using energy dispersive X‐ray spectroscopy (EDX) in STEM spectrum imaging mode. The EDX spectra were collected using a four‐silicon drift detector (SDD) system (Super X detector, Thermo Fisher Scientific). The beam current for STEM‐EDX analysis was ≈300 pA. UV–vis absorption spectra were measured on quartz substrates with a PerkinElmer Lambda 1050 spectrometer with an integrating sphere. SEM characterization was performed on a tungsten thermionic emission SEM (TESCAN VEGA).

### Electrical Measurements

All electrical characterizations of 2D transistors were performed in a vacuum (≈10^−4^ Pa) probe station using Keithley 2450, Keithley 2636B, and Agilent 2912A source meters at room temperature. Flexible top‐gated MoS_2_ transistors were initially measured in vacuum probe station to establish performance benchmark of the intrinsic flat state. Subsequent in situ bending tests were performed in a home‐built probe station inside a nitrogen‐filled glove box at ambient pressure (Figure S9, Supporting Information).

## Conflict of Interest

The authors declare no conflict of interest.

## Author Contributions

Z.G., X.S., and F.X. contributed equally to this work. W.S. and H.G. conceived and supervised the project. F.X., J.Z., and L.W. grew the freestanding BTO membranes. Z.G., X.S., G.Z., J.L., Q.L., and W.S. fabricated the devices and performed electrical measurements. G.H. and C.Z. conducted the TEM characterization. Z.G., X.S., C.Z., H.H., L.W., H.G., and W.S. analyzed the data; Z.G. and Y.M. carried out the ANN simulations. W.S., J.S., H.G., Z.G., and X.S. wrote the manuscript with input from all authors.

## Supporting information



Supporting Information

## Data Availability

The data that support the findings of this study are available from the corresponding author upon reasonable request.
