# Supplementary material for: Integration of Freestanding High‐k Oxide Membranes for 2D Ferroelectric Field‐Effect Transistors
Source: Adv Sci (Weinh). 2025 Dec 14;13(12):e20610. doi: 10.1002/advs.202520610 (PMC12948196; doi:10.1002/advs.202520610)
Supplement: Supplementary file 1 — Supporting Information [file ADVS-13-e20610-s001.pdf]

Supporting Information

Integration of Freestanding High-*k* Oxide Membranes for Two-Dimensional Ferroelectric Field-Effect Transistors

Zejing Guo, Xuyang Sha, Fang Xu, Guangyi Huang, Jinfeng Zhang, Yang Mou, Guorui Zhao, Jiaqi Liu, Qing Lan, Wenqing Song, Cheng Zhang, Hai Huang, Changlin Zheng, Lingfei Wang, Hangwen Guo, Jian Shen*, Wu Shi**

Z. Guo, X. Sha, F. Xu contributed equally to this work.
E-mail: hangwenguo@fudan.edu.cn; shenj5494@fudan.edu.cn; shiwu@fudan.edu.cn

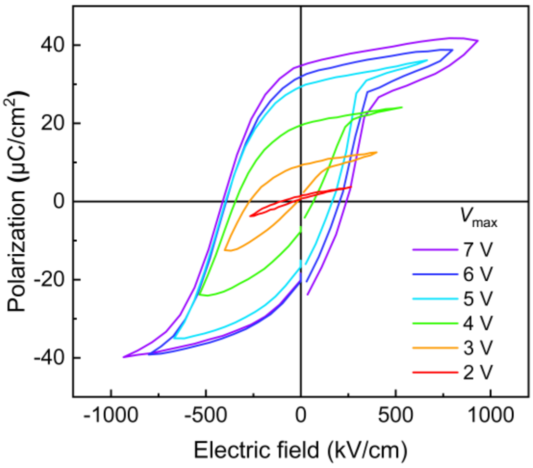

Figure S1. Polarization-electric field (*P*-*E*) hysteresis loops of 75 nm $BaTiO_3$ (BTO) measured at 1 kHz as a function of sweeping voltage ranges. In the *P*-*E* measurements, voltage of a triangular waveform was applied to a representative capacitor with Pt/BTO/LSMO (20 nm)/ STO (001) to obtain the polarization hysteresis loops. A large remnant polarization (~34 μC/cm^2) was displayed.

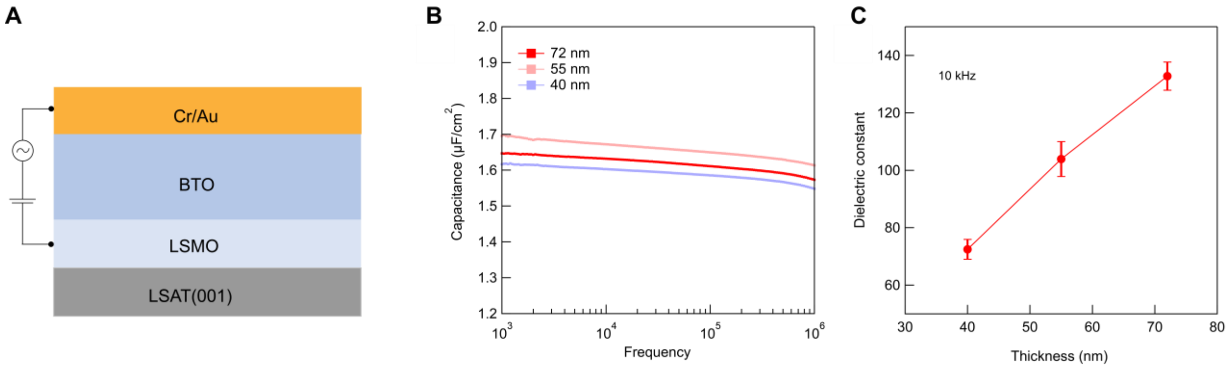

Figure S2. Dielectric properties of as-grown epitaxial BTO films with different thicknesses. A) Schematic of the parallel-plate capacitors with vertical MIM structure. B) Frequency-dependent capacitance (*C*–*f*) measured from large-area Au/BTO/LSMO/LSAT capacitors with BTO thicknesses of 40 nm, 55 nm, and 72 nm. C) Extracted dielectric constant as a function of BTO thickness. A clear monotonic increase of *ε* with thickness is observed, consistent with the "dead layer" model.[1]

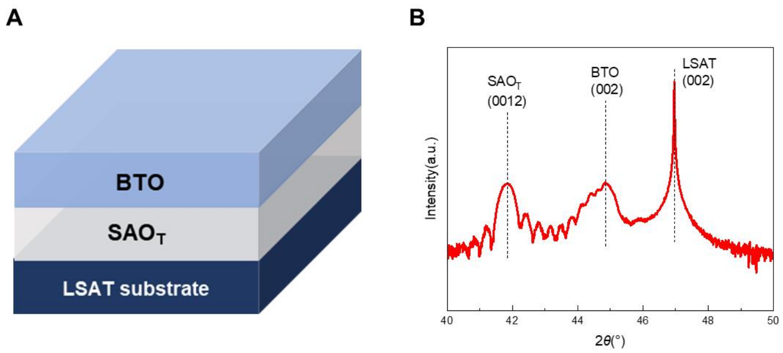

Figure S3. XRD characterization for BTO/SAO$_T$/LSAT (001) heterostructures. A) Schematic of the as-grown BTO/SAO$_T$/LSAT (001) heterostructure before releasing the BTO membrane from the sacrificial SAO$_T$ layer. B) 2θ-ω XRD diffraction scan of BTO/SAO$_T$/LSAT (001) heterostructure. The labeled peak positions confirm the single crystalline nature of the as-grown BTO/SAO$_T$/LSAT (001) heterostructure, and the observed Laue fringes indicate the film's ultra-high quality.

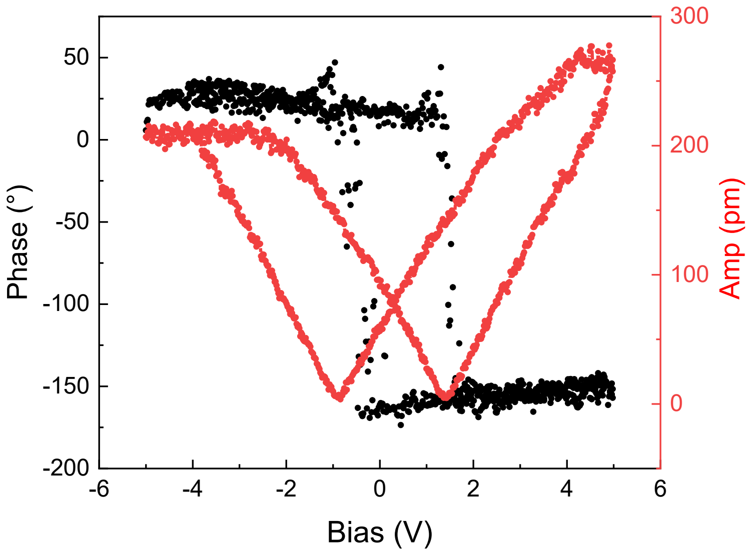

Figure S4. Local phase and amplitude hysteresis loops of a freestanding BTO membrane**.** The hysteresis loops were recorded with a 500 mV AC bias applied to the scanning tip during the PFM measurements. The 180° out-of-plane phase hysteresis and butterfly-shaped amplitude loop confirm the out-of-plane ferroelectric behavior of the BTO membrane.

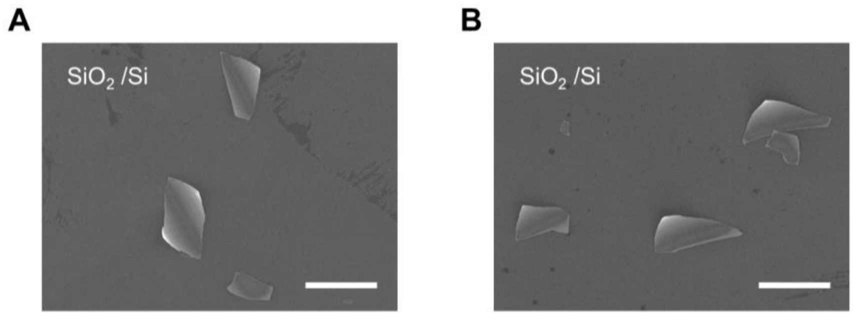

Figure S5. SEM images of curled BTO membranes. A) and B) SEM images of curled BTO membranes directly transferred onto the SiO_2 / Si substrate by a standard dry-transfer method, demonstrating the intrinsic elasticity of the freestanding BTO membranes. Scale bar, 10 µm.

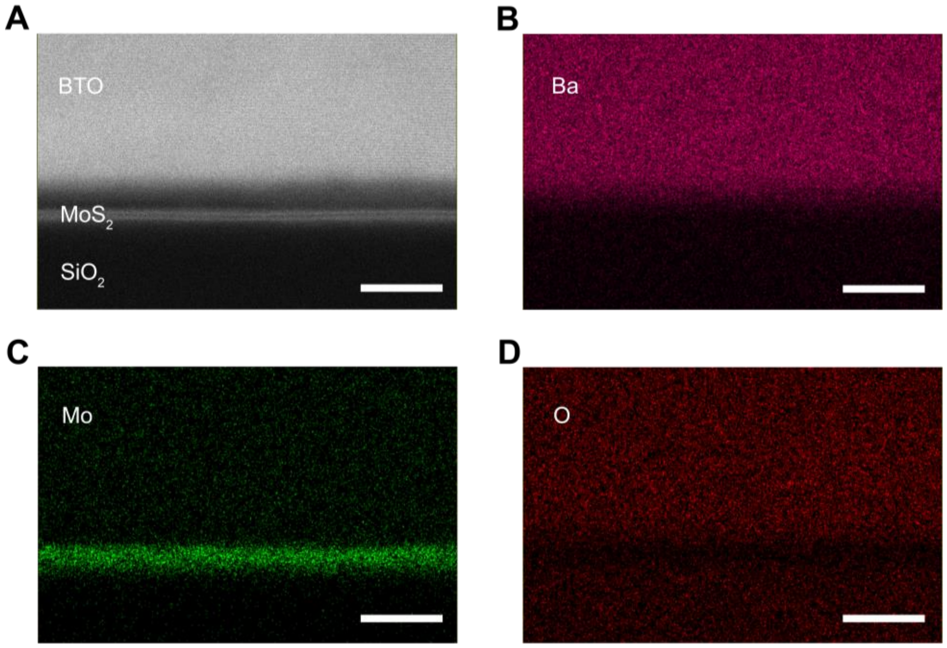

Figure S6. STEM characterizations of the top-gated MoS_2 FET. A) Cross-sectional HAADF-STEM image of a BTO/bilayer MoS_2 transistor in the channel region. STEM-EDS mapping of (B) barium, (C) molybdenum and (D) oxygen elements. Scale bar, 10 nm.

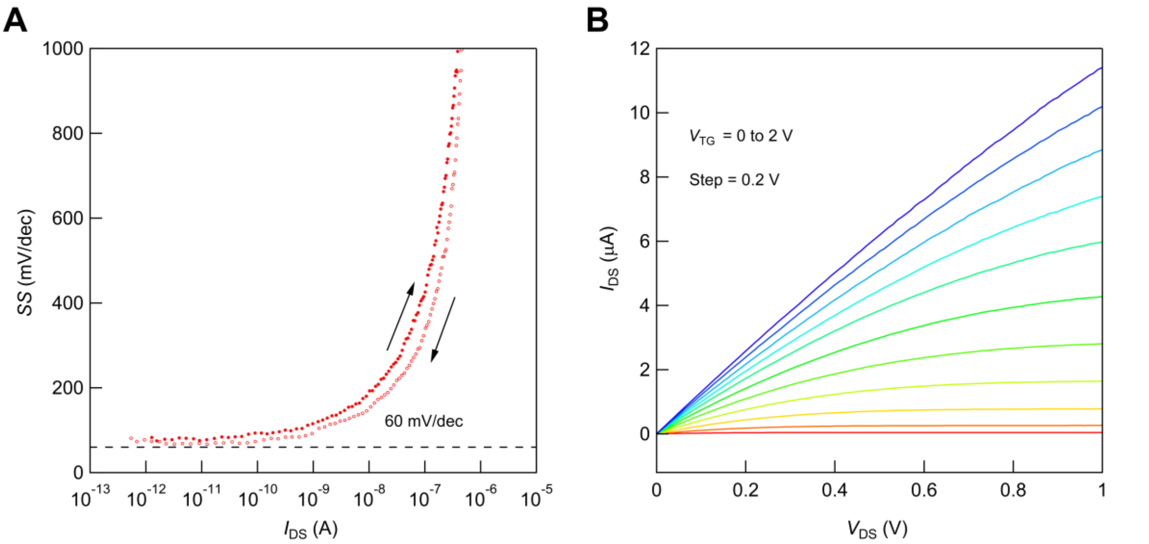

Figure S7. Electrical characteristics of the BTO/MoS_2 transistor shown in Figure 2B. A) Subthreshold swing (SS) of the MoS_2 transistor as a function of I_{DS}, extracted from Figure 2B. The dashed line indicates the theoretical limit of 60 mV/dec. Notably, the SS remains consistently low across several orders of magnitude in I_{DS}, highlighting the strong gate modulation capability of freestanding BTO for low-voltage operation. B) Output characteristics of the same MoS_2 transistor. The output curve exhibits a linear response at low source-drain bias V_{DS} and saturation at high V_{DS}, indicating ohmic contacts and effective current modulation.

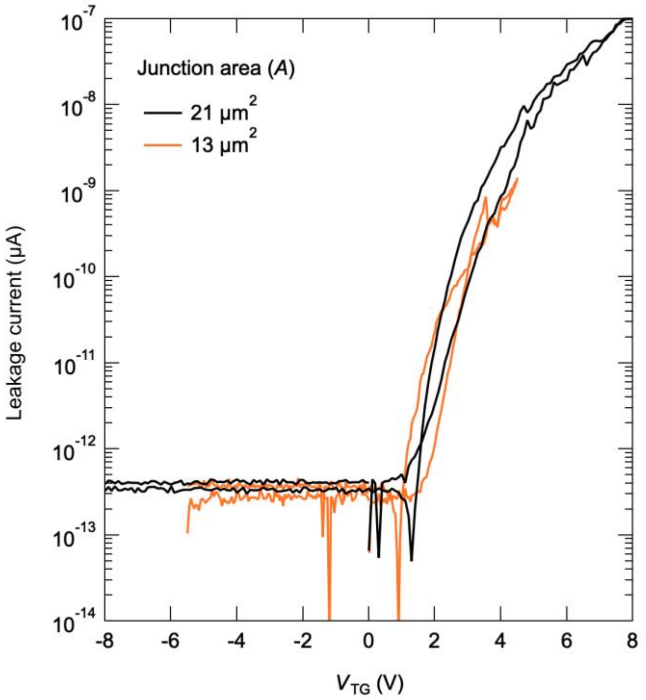

Figure S8. Leakage current versus top-gate voltage V_{TG} for BTO/MoS_2 transistors with large dielectric-channel junction areas. These two transistors use 75 nm and 40 nm BTO membranes

as the top-gate dielectric, with junction areas of 21 μm^2 and 13 μm^2, respectively. Both show a marked increase in leakage currents above V_{TG} = 2 V, likely caused by oxygen vacancy defects in the BTO layer and trap states at the MoS_2/BTO interface.[2-4] This underscores leakage current as a common challenge for BTO-integrated 2D transistors, restricting their operational voltage range and limiting the effective polarization reversal. In this work, we develop a defect-tolerant strategy that substantially reduces the dielectric-channel junction area, thereby enabling robust ferroelectric switching in the BTO-integrated 2D transistors (Figure 2C-E).

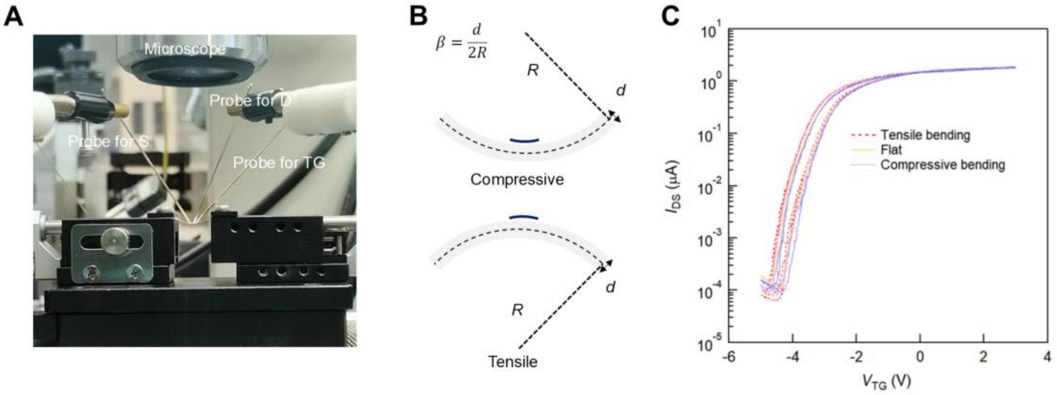

Figure S9. Characterization of a flexible BTO/MoS_2 transistor under tensile and compressive bending. A) Optical image of a BTO/MoS_2 transistor on a flexible mica substrate, measured under compressive bending in a home-built probe station inside a nitrogen-filled glove box at ambient pressure. B) Schematic illustration of the compressive (top) and tensile (bottom) bending states. The applied strain is given by $d/2R$, where d is the mica thickness and R is the bending radius. C) Transfer characteristics of the flexible BTO/MoS_2 transistor under different three conditions: the pristine flat state (orange solid line), tensile bending (red dashed lines) with a maximum strain of ~0.21%, and compressive bending (blue solid lines) with a maximum strain of ~0.15%. The device maintains consistent performance under all bending states, demonstrating its mechanical flexibility.

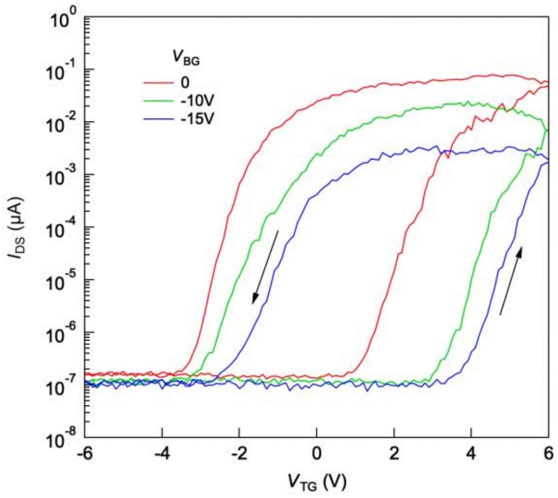

Figure S10. Transfer curves of a BTO-MoS_2 FeFET under various back-gate voltages V_{BG}. Across all V_{BG} conditions, the FeFET exhibits pronounced anticlockwise hysteresis. As V_{BG} decreases, the *n*-type MoS_2 channel becomes less conductive, causing a rightward shift in the top-gate transfer curve and the memory window. This behavior highlights the tunability of the memory window in our dual-gate ferroelectric transistors.

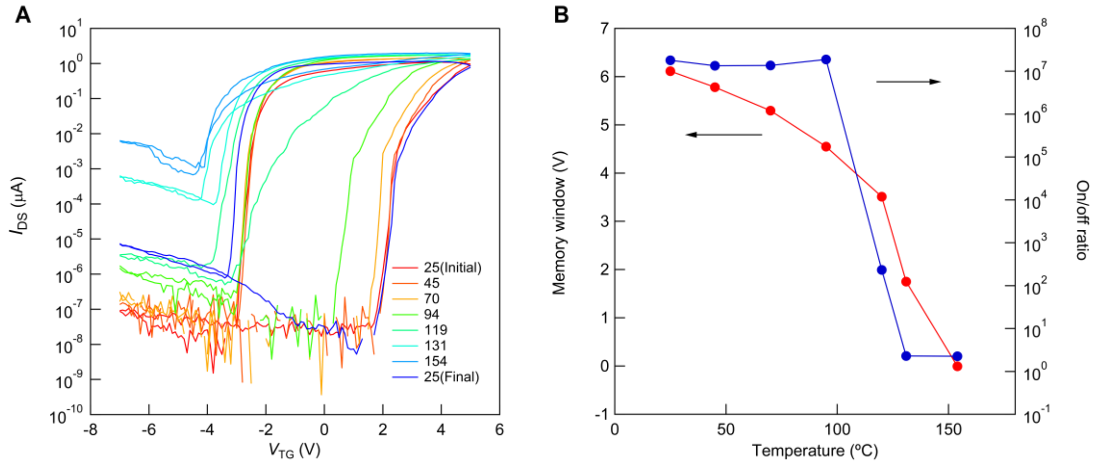

Figure S11. Temperature-dependent electric properties of the BTO/MoS_2 FeFET. A) Temperature-dependent transfer curves of BTO/MoS_2 FeFET. The hysteresis gradually narrows with increasing temperature and recovers when cooling to room temperature. While thermally activated defects contribute to gradual degradation and increased off-state current, the reversible hysteresis indicates that the memory window reduction is primarily driven by the ferroelectric-to-paraelectric phase transition. [5,6] B) Memory window and on/off ratio of BTO/MoS_2 FeFET under elevated temperature.

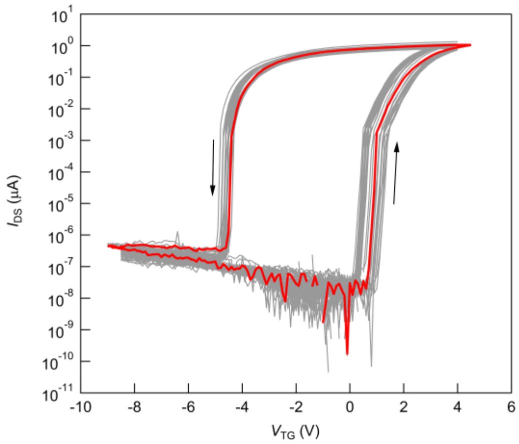

Figure S12. Cycling test for a 40 nm BTO-based FeFET. The test was conducted over numbers of operation cycles within consistent sweeping range. A large memory window of 50% is stable over 40 operation cycles.

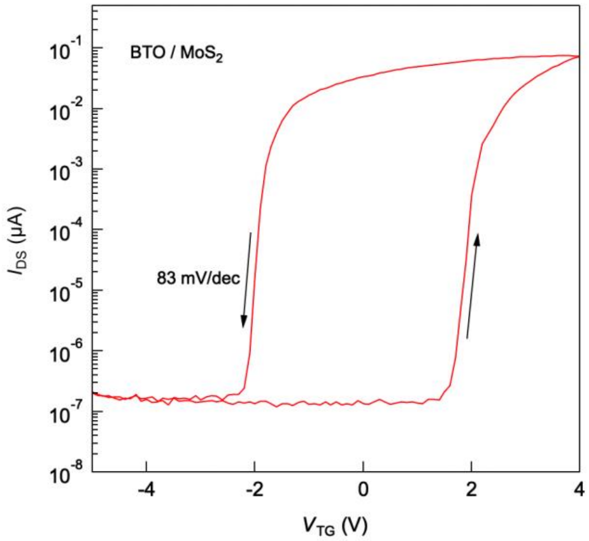

Figure S13. Transfer curve of a 55 nm-BTO/MoS_2 FeFET. The device exhibits an on/off ratio approaching 10^6, a minimal SS of 83 mV/dec, and a memory window over 47 % of the scan voltage range.

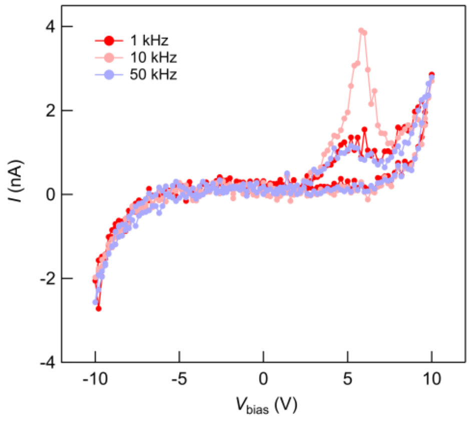

Figure S14. Leakage current–voltage characteristics of a 72 nm BTO junction with Bi/Au top electrodes and Pt bottom electrodes. A pronounced leakage peak is observed at ~5 V, corresponding to the onset of ferroelectric switching in the junction. The disappearance of current peak at negative voltage could be explained by electrode asymmetry shifts the switching threshold differently for the two sweep directions.

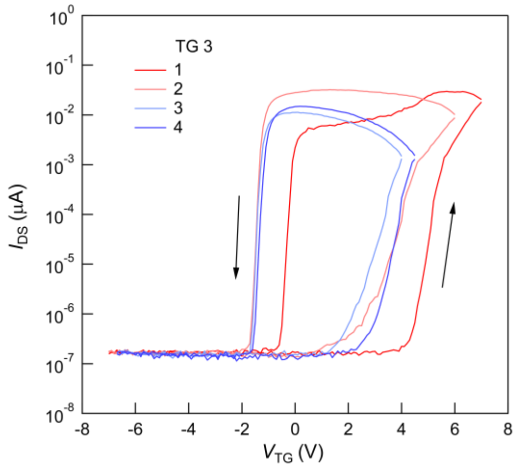

Figure S15. Transfer curves of four memory cells that share the third top gate in Figure 4A.

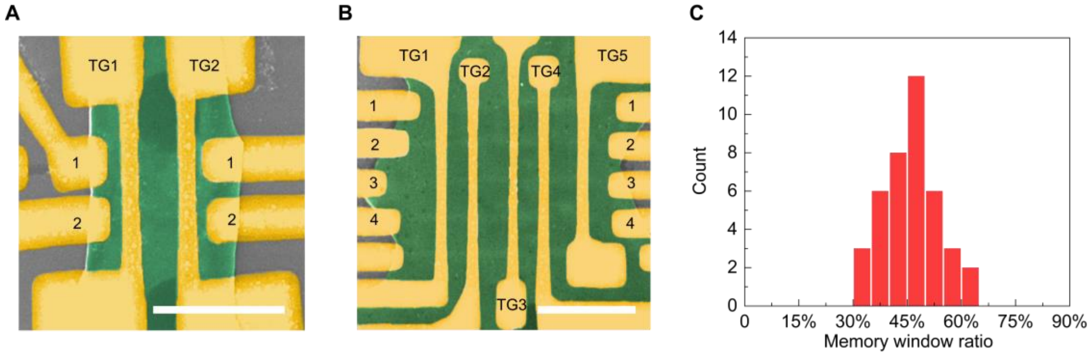

Figure S16. Device fabrication and performance consistency. A) and B) False-color SEM images of two additional representative FeFET arrays, demonstrating repeatable device

fabrication beyond the sample presented in the main text. Scale bar, 5µm. C) Statistical distribution of the memory window (MW) ratio compiled from 40 functional devices.

Ferroelectric materials	Thickness (nm)	On/off ratio	MW ratio	Normalized MW(V/nm)	Flexibility
PZT[7]	100	10^1	0.333	0.04	-
HZO[8]	15	10^5	0.091	0.0667	-
CIPS[9]	400	10^1	0.545	0.015	-
P(VDF-TrFE) [10]	300	10^6	0.421	0.107	√
PMN-PT[11]	$5x10^5$	10^6	0.333	$2x10^{-4}$	-
AlScN[12]	100	10^7	0.525	0.21	-
BTO (this work)	**40**	$\mathbf{10^6}$	**0.541**	**0.22**	√

Table S1. Performance comparison of 2D FeFETs with metal-ferroelectric-semiconductor architectures.

References

1. M.Stengel, N. Spaldin, Origin of the dielectric dead layer in nanoscale capacitors. *Nature* **443**, 679–682 (2006).
2. J. Choi, K. J. Crust, L. Li, K. Lee, J. Luo, J.-P. So, K. Watanabe, T. Taniguchi, H. Y. Hwang, K. F. Mak, J. Shan, G. D. Fuchs, Tuning Exciton Emission via Ferroelectric Polarization at a Heterogeneous Interface between a Monolayer Transition Metal Dichalcogenide and a Perovskite Oxide Membrane. *Nano Lett* **24**, 8948–8955 (2024).
3. T. Pucher, S. Puebla, V. Zamora, E. Sánchez Viso, V. Rouco, C. Leon, M. Garcia-Hernandez, J. Santamaria, C. Munuera, A. Castellanos-Gomez, Strong electrostatic control of excitonic features in MoS_2 by a free-standing ultrahigh-κ ferroelectric perovskite. *Adv Funct Mater* **34**, 2409447 (2024).
4. P. Debashis, H. Ryu, R. Steinhardt, P. Buragohain, J. J. Plombon, K. Maxey, K. P. O'Brien, R. Kim, A. Sen Gupta, C. Rogan, J. Lux, I.-C. Tung, D. Adams, M. E. Gulseren, A. Verma Penumatcha, S. Shivaraman, H. Li, T. Zhong, S. Harlson, T. Tronic, A. Oni, S. Putna, S. B. Clendenning, M. Metz, M. Radosavljevic, U. Avci, I. A. Young, Ultra-high- *k* ferroelectric $BaTiO_3$ perovskite in the gate stack for two-dimensional WSe_2 p-type high-performance transistors. *Nano Lett* **24**, 12353–12360 (2024).
5. S. Puebla, T. Pucher, V. Rouco, G. S.-Santolino, Y. Xie, V. Zamora, F. A. Cuellar, F. J. Mompean, C. Leon, J. O. Island, M. G.-Hernandez, J. Santamaria, C.Munuera, and A. C.-Gomez, Combining Freestanding Ferroelectric Perovskite Oxides with Two-Dimensional Semiconductors for High Performance Transistors. *Nano Lett* **22**, 7457 (2022).
6. H.Sun, P.Chen, W.Mao, C.Guo, Y.Li, J.Wang, W.Sun, D.Xu, B.Hao, T.Zhang, J.Ma, J.Yang, Z.Cao, S.Yan, Y.Guan, Z.Wen, Z.Mao, N.Zheng, Z.Gu, H.Huang, P.Wang, Y.Zhang, D.Wu & Y.Nie, Ferroelectric topologies in $BaTiO_3$ nanomembranes for light field manipulation. *Nat. Nanotechnol.* **20**, 881–888 (2025).
7. A. Lipatov, P. Sharma, A. Gruverman, A. Sinitskii, Optoelectrical molybdenum disulfide (MoS_2)—ferroelectric memories. *ACS Nano* **9**, 8089–8098 (2015).
8. K. Toprasertpong, K. Tahara, T. Fukui, Z. Lin, K. Watanabe, M. Takenaka, S. Takagi, Improved ferroelectric/semiconductor interface properties in $Hf_{0.5}Zr_{0.5}O_2$ ferroelectric FETs by low-temperature annealing. *IEEE Electron Device Letters* **41**, 1588–1591 (2020).
9. M. Si, P.-Y. Liao, G. Qiu, Y. Duan, P. D. Ye, Ferroelectric Field-Effect Transistors Based on MoS_2 and $CuInP_2S_6$ Two-Dimensional van der Waals Heterostructure. *ACS Nano* **12**, 6700–6705 (2018).

10. L. Liu, X. Wang, L. Han, B. Tian, Y. Chen, G. Wu, D. Li, M. Yan, T. Wang, S. Sun, H. Shen, T. Lin, J. Sun, C. Duan, J. Wang, X. Meng, J. Chu, Electrical characterization of MoS_2 field-effect transistors with different dielectric polymer gate. *AIP Adv* **7**, 065121 (2017).

11. L. Xu, Z. Duan, P. Zhang, X. Wang, J. Zhang, L. Shang, K. Jiang, Y. Li, L. Zhu, Y. Gong, Z. Hu, J. Chu, Ferroelectric-modulated MoS_2 field-effect transistors as multilevel nonvolatile memory. *ACS Appl Mater Interfaces* **12**, 44902–44911 (2020).

12. K.-H. Kim, S. Oh, M. M. A. Fiagbenu, J. Zheng, P. Musavigharavi, P. Kumar, N. Trainor, A. Aljarb, Y. Wan, H. M. Kim, K. Katti, S. Song, G. Kim, Z. Tang, J.-H. Fu, M. Hakami, V. Tung, J. M. Redwing, E. A. Stach, R. H. Olsson, D. Jariwala, Scalable CMOS back-end-of-line-compatible AlScN/two-dimensional channel ferroelectric field-effect transistors. *Nat Nanotechnol* **18**, 1044–1050 (2023).
